# Advances in Synthetic Fluorescent Probe Labeling for Live-Cell Imaging in Plants

**DOI:** 10.1093/pcp/pcab104

**Published:** 2021-07-18

**Authors:** Noriyoshi Yagi, Akira Yoshinari, Ryu J Iwatate, Reika Isoda, Wolf B Frommer, Masayoshi Nakamura

**Affiliations:** Institute of Transformative Bio-Molecules (WPI-ITbM), Nagoya University, Chikusa, Nagoya 464-8601, Japan; Institute of Transformative Bio-Molecules (WPI-ITbM), Nagoya University, Chikusa, Nagoya 464-8601, Japan; Institute of Transformative Bio-Molecules (WPI-ITbM), Nagoya University, Chikusa, Nagoya 464-8601, Japan; School of Medicine, Nagoya University, Universitätsstr. 1, Showa, Nagoya 466−8550, Japan; Institute of Transformative Bio-Molecules (WPI-ITbM), Nagoya University, Chikusa, Nagoya 464-8601, Japan; Institute of Transformative Bio-Molecules (WPI-ITbM), Nagoya University, Chikusa, Nagoya 464-8601, Japan; Institute for Molecular Physiology, Heinrich-Heine-University Düsseldorf, Düsseldorf 40225, Germany; Institute of Transformative Bio-Molecules (WPI-ITbM), Nagoya University, Chikusa, Nagoya 464-8601, Japan

**Keywords:** Fluorescent probes •, Live-cell imaging •, Self-labeling protein tag •, SNAP-tag •, Synthetic dyes

## Abstract

Fluorescent probes are powerful tools for visualizing cellular and subcellular structures, their dynamics and cellular molecules in living cells and enable us to monitor cellular processes in a spatiotemporal manner within complex and crowded systems. In addition to popular fluorescent proteins, a wide variety of small-molecule dyes have been synthesized through close association with the interdisciplinary field of chemistry and biology, ranging from those suitable for labeling cellular compartments such as organelles to those for labeling intracellular biochemical and biophysical processes and signaling. In recent years, self-labeling technologies including the SNAP-tag system have allowed us to attach these dyes to cellular domains or specific proteins and are beginning to be employed in plant studies. In this mini review, we will discuss the current range of synthetic fluorescent probes that have been exploited for live-cell imaging and the recent advances in the application that enable genetical tagging of synthetic probes in plant research.

## Introduction

Over the past decades, a wide range of fluorescent dyes have been synthesized and deployed for addressing cellular biological questions in plant sciences. There has also been a continuous effort by chemists to improve the characteristics and applications of dyes. Many of the original dyes can only be used in fixed tissues since they do not permeate into live cells. Still, they are frequently used to mark or highlight organelles in multicolor imaging approaches. With the advent of fluorescent proteins (FPs), many scientists have shifted to using FPs that can be genetically targeted, not just to specific organelles but to even specific subdomains by selecting suitable FPs (Cutler et al. 2000). These approaches are advantageous for example when attempting to identify subpopulations of vesicles or subdomains of the plasma membrane (PM). A wide range of spectral variants of FPs have been made available, and their properties such as maturation time and quantum yield are being continuously improved (Tsien 1998, Frommer et al. 2009, Isoda et al. 2021). Even then, FPs can have intrinsic disadvantages; they can affect the targeting of the protein of interest (POI) due to their large size, their maturation time can affect interpretation of the data, and the photostability tends to be relatively limited. Covalent self-labeling technology provides a way to combine synthetic dyes with genetical tagging, thereby providing access to the large realm of synthetic dyes that have superiority in terms of size and photostability. All three approaches are complementary and may be used either simultaneously in multicolor approaches, or to control for possible artifacts introduced by the specific approaches. Here we provide an overview of synthetic small-molecule-based fluorescent probes that are applied to live imaging with subcellular resolutions and recent successful examples of covalent self-labeling of tagged protein with fluorescent probes in plants.

## Fluorescent Dyes for Live Imaging of Cellular Compartments

A wide range of small-molecule synthetic fluorescent dyes have been developed for live imaging (Zhu et al. 2016). These fluorophores localize to certain cell populations and/or organelles. In this review, we provide examples of dyes that have been used successfully to label specific cellular compartments or subdomains and functional dyes for cellular activities and viability. Application of these synthetic fluorescent dyes allow for rapid and highly efficient labeling in vivo and can be used complementarily or simultaneously with FP-tagged POIs for subcellular localization and multicolor imaging. Dyes for studying cell–cell connectivity and for visualizing cell–cell translocation of plant hormone have also been developed and reported (Oparka et al. 1994, Rigal et al. 2014), but they will not be discussed here. The selective localization to compartments relies on the specific chemical properties of the dyes, e.g. lipophilicity and charge. **Table 1** provides an overview of the different dyes and their uses. Moreover, we discuss the possibility of attachment of fluorescent dyes to specific proteins using self-labeling technology to form covalent bonds, which has recently been shown to be readily applicable in plant sciences (Iwatate et al. 2020).

**Table 1:** Synthetic dyes for live plant cell imaging

Indicator	Ex/Em	Target	Plant material tested	Reference
Fluorescent dyes for live imaging of cellular components				
FM®1-43	488/505–550	PM, endosome and tonoplast	Arabidopsis root and tobacco BY-2 cells	Jelínková et al. (2010), Feraru et al. (2010), Scheuring et al. (2016)
FM®4-64	488 or 514 or 561/>570			
SP-468	490/502–585	PM	Tobacco seedling	Collot et al. (2020)
DilC_12_(3)	543/560–620	Membrane compartment	Arabidopsis protoplast	Blachutzik et al. (2012)
DilC_18_(3)	543/560–620			
LRB-PE	543/580–630	Phospholipid-enriched membrane compartment		
BD-SM	488/500–550	Sphingolipid-enriched membrane compartment		
Laurdan	Ratiometric (760**/400–440 or 490–550)	Membrane lipid phase		
Di-4ANEPPDHQ	Ratiometric (488/500–580 or 620–750)		Arabidopsis root	Zhao et al. (2015)
ER-Tracker Blue-White DPX	405/425–475	ER	Populus leaf and Arabidopsis pollen tube	Takata and Eriksson (2012), Wang et al. (2015)
ER-Tracker™ Red	561/600–650		Arabidopsis root	Yoshinari et al. (2020)
BODIPY® TR C_5_-ceramide	532/640–660	Golgi apparatus	Populus leaf, Arabidopsis and tobacco pollen tube, and Arabidopsis culture cell	Takata and Eriksson (2012), Wang et al. (2015)
MDY-64	458/465–550	Tonoplast	Arabidopsis root	Scheuring et al. (2015), Scheuring et al. (2016)
MitoTracker™ Orange (CMTMRos)	543/540–600	Mitochondria	Arabidopsis leaf	Arimura et al. (2008)
DiOC_6_(3)	488/503–523		Moss living buds and tobacco plant	Matzke and Matzke (1986), Martens et al. (2006)
HoeAc_2_Fl	470–490/515–550	DNA (nuclei/chromosomes)	Arabidopsis stomata	Takaoka et al. (2020)
SYTO® 12	499/522		Maize meiotic cells	Nannas et al. (2016)
SYTOX® blue	458/475–500	DNA (nonviable cells)	Arabidopsis embryo and root	Truernit and Haseloff (2008)
SYTOX® green	488/510–560			
SYTOX® orange	568/580–610			
Propidium iodide	495/>565	Cell wall (homogalacturonans)	Arabidopsis pollen tube and Arabidopsis root hair	Rounds et al. (2011)
Pontamine Fast Scarlet 4B (Direct Red 23)	561/>575	Cellulose	Arabidopsis seedling	Anderson et al. (2010)
Aniline blue	405/439–484	Callose	Arabidopsis and tobacco leaf	Kraner et al. (2016)
Kdo-N_3_ and alkyne-containing fluorescent probe	Depends on dyes used	RG-II	Arabidopsis seedling and BY-2 cells	Dumont et al. (2016)
Fluorescence-tagged monolignols	Depends on dyes used	Lignin	Arabidopsis seedling, stems and mesophyll protoplast	Tobimatsu et al. (2013)
Dyes for monitoring biochemical and biophysical processes and signaling				
H_2_DCFDA and CM-H_2_DCFDA	488/490–606	ROS	Tobacco leaf, Arabidopsis root hair and Arabidopsis whole plant (leaves)	Govrin and Levine (2000), Foreman et al. (2003), Fichman et al. (2019)
OxyBURST® Green H_2_HFF BSA	488/>505		Arabidopsis root hair and Arabidopsis whole plants (leaves)	Monshausen et al. (2007), Fichman et al. (2019)
PO1	488/544–624		Arabidopsis whole plants (leaves)	Fichman et al. (2019)
BCECF	Ratiometric (490 or 450/520)	pH change	Lily pollen tubes and Arabidopsis root cap cells	Feijo et al. (1999), Fasano et al. (2001)
HPTS	Ratiometric (405 or 458/499–546)		Arabidopsis seedling	Barbez et al. (2017), Dünser et al. (2019)
SNARF®-1	Rariometric (488/540–590 or 610–670)		BY-2 protoplast and Arabidopsis root	Sano et al. (2010), Viotti et al. (2013)
Oregon Green® 488	Ratiometric (480 or 440/>520)		Arabidopsis root	Fasano et al. (2001)
DiBAC_4_(3)	480/510–560	Membrane potential	Vicia guard cell protoplast, Arabidopsis mesophyll protoplast, stem epidermal cells and root epidermal cells	Konrad and Hedrich (2008), Lu et al. (2016), Dejonghe et al. (2016), Hoffmann et al. (2020)
ANNINE-6 plus	475/499–549 and 573–627		Arabidopsis pollen tubes	Hoffmann et al. (2020)
sulfo-BDP, polyethylene glycol-BDP, N^+^-BDP and cell wall binding peptide-BDP		Microviscosity	Arabidopsis root, cotyledon and cultured cells	Michels et al. (2020)

**Two-photon imaging

### Plasma membrane/Endosome/Lipid

Fei Mao (FM) dyes are styryl dyes with amphiphilic characteristics that are widely used to stain PMs. Among FM dyes, FM4-64 is one the most popular dyes in the realm of plant live-cell imaging, because of its chemical stability and wavelength [excitation/emission maxima (Ex/Em), 515/640 nm] which enable it to be used for live imaging with green/yellow FPs (Bolte et al. 2004). FM1-43, one of the derivatives of FM4-64, has shorter Ex/Em (479/598 nm) and is also widely used to label PMs of plants, including BY-2 cells (Bolte et al. 2004). Due to the nature of the membrane-bound dye, side effects on the labeled membrane and the membrane-localized protein functions have been reported (Jelínková et al. 2010); hence, the handling and the data interpretation should be done with care. PMs are internalized via endocytosis and PM-derived lipids are incorporated into the membranes of the *trans*-Golgi network/early endosome (TGN/EE). FM dyes, which initially label the PM, are internalized over time and can be utilized to assess endocytic activities (Feraru et al. 2010). Collot et al. (2020) developed the FM1-43 derived dyes SP-468 and SQ-535, which have higher photostability compared to the original. SP-468, in particular, was shown to be suitable for stimulated emission depletion super-resolution imaging.

PMs are mainly composed of three types of lipids: phospholipids, sphingolipids and sterols. These lipids are not uniformly distributed but heterogeneously spread throughout the PM. Lipophilic carbocyanine dyes such as dioctadecyl-tetramethylindocarbocyanines (‘dye-aye’;DiIs) partitioning preferentially in the model membrane systems (Juhasz et al. 2010) have been employed in plant cells. DiIC_18_(3) and DiIC_12_(3) (Ex/Em, 549/565 nm) have been reported to heterogeneously label Arabidopsis protoplast PMs (Blachutzik et al. 2012). The lipid-conjugated probes Lissamine rhodamine B labeled phosphatidyl ethanolamine (LRB-PE) and BODIPY FL C_12_-sphingomyelin (BD-SM) have also been used for protoplast membrane lipid phase analysis where LRB-PE (Ex/Em, 560/583 nm) and BD-SM (Ex/Em, 505/515 nm) stained phospholipid-enriched and sphingolipid-enriched compartments, respectively, and both dyes were retained at PM longer than DiI dyes in Arabidopsis protoplasts (Blachutzik et al. 2012). According to the spectra of the probe, BD-SM can be utilized for the simultaneous staining with other membrane dyes such as FM4-64. The solvatochromic dye, Laurdan, has also been shown to be incorporated into the PM and applicable for lipid phase imaging of Arabidopsis protoplasts (Blachutzik et al. 2012), which demanded an employment of two-photon microscopy. Another solvatochromic dye di-4-ANEPPDHQ changes its fluorescence spectrum depending on the membrane environment, which allows us to quantitatively visualize the membrane orders in live plant cells. Live-cell imaging of lipid phases using di-4-ANEPPDHQ revealed that the membrane organization in the PM is higher than that in endomembrane systems in growing root hair cells, although it requires spectral imaging systems and accurate image analysis in which the specimen was excited at a wavelength of 488 nm and the fluorescence was collected through two channels (500–580 nm and 620–750 nm) (Zhao et al. 2015).

### Endoplasmic reticulum/Golgi apparatus/Vacuole

The endoplasmic reticulum (ER) is a membrane network that exhibits dynamic movements and shape transitions within the cell. ER-Tracker™, a fluorescent ER probe, consists of a fluorophore and a drug-derived moiety called glibenclamide that binds to adenosine triphosphate (ATP)-sensitive K^+^ channels present in the ER. ER-Tracker™ probes have been used in dynamics analyses to stain the ER in pollen tubes (Wang et al. 2015) and root epidermal cells of Arabidopsis (Yoshinari et al. 2020). However, it is worth noting that glibenclamide is an inhibitor of ATP-sensitive potassium channels (Martin et al. 2017), so ER-Tracker™ may interfere with normal ER activity.

Fluorophore-conjugated ceramides are actively incorporated into Golgi membranes and label the Golgi apparatus. BODIPY TR C_5_-ceramide (Ex/Em, 589/616 nm) was used to label the Golgi apparatus of Arabidopsis and tobacco pollen tubes (Wang et al. 2015) and leaf epidermal cells in *Populus* (Takata and Eriksson 2012). BODIPY FL C_5_-ceramide stained Golgi apparatus more rapidly than BODIPY TR C_5_-ceramide in COS7 cells (Tóth et al. 2006); however, in plant cells BODIPY FL C_5_-ceramide showed distinct localization pattern (Cutler et al. 2000).

Vacuoles can be visualized by staining the tonoplast (vacuolar membrane) or the vacuolar lumen. In plant cells, PM lipids are transported to the tonoplast via endosomes including TGN/EE and multi-vesicular bodies/late endosome. Therefore, the dyes used to stain PM such as FM4-64 and FM1-43 can be used to image vacuoles (Feraru et al. 2010, Jelínková et al. 2010). However, long-term uptake (starting 2–3 hours after application) is required to accumulate the dye at the tonoplast, and the study by Jelínková et al. (2010) also showed that dyes can affect cellular processes such as auxin transporter trafficking and thus results need to be interpreted with caution. A green fluorescent tonoplast marker MDY-64 (Ex/Em, 451/497 nm) has also been utilized for imaging plant cells (Scheuring et al. 2015). Fluorescence of MDY-64 is also seen at the cell exterior such as the cell wall, but reliable fluorescence appears in the tonoplast within 5 min after application. The pH-sensitive fluorescent dye 2ʹ,7ʹ-*bis*-(2-carboxyethyl)-5-(and-6)-carboxyfluorescein, acetoxymethyl ester (BCECF-AM), which accumulates in the vacuolar lumen, allows the 3D reconstruction of the vacuole and the analysis of its volume and morphology (Kalinowska et al. 2015, Scheuring et al. 2016).

### Mitochondria/Nuclei

As primary sources of ATP and redox potential production, mitochondria play a pivotal role in all plant cells. Mitochondria are motile and highly dynamic in their shape and cellular distribution. The MitoTracker™ series, which contains chloromethyl moieties mediating the binding of free sulfhydryl groups, are well-known probes commercially available for mitochondria visualization and have successfully been used for live imaging of plant mitochondria (e.g. Arimura et al. 2008). 3,3ʹ-dihexykloxacarbocyanine iodide [DiOC_6_(3)], a cyanine derivative dye, was also used to visualize plant mitochondria (Matzke and Matzke 1986), but ER was also labeled when applied at the higher concentrations (Martens et al. 2006).

Nuclei have been labeled widely using dyes such as 4ʹ,6-diamidino-2-phenylindole (DAPI) or Hoechst 33342 (Latt et al. 1975, Kapuscinski 1995), but there are only a few reports of synthetic dyes for staining the nuclei of living plant cells. The use of DNA fluorescent probes in living plant cells remains questionable. When incubated for 60 min in the presence of 50 µg/ml DAPI, the majority (74%) of unfixed petunia protoplasts did not fluoresce and the remaining 26% of the protoplasts showed fluorescence but the fluorescence was not sufficient to classify the G1 phase of the cell cycle (Kamo and Griesbach 1993). Also, DAPI and other DNA dyes (Hoechst 33258, Hoechst 33342, propidium iodide, SYTO11 and SYTO13-17) did not stain the chromosomes in cultured maize meiocytes (Yu et al. 1997). The development and investigation of dyes that permeate PMs in plant cells have progressed, and recently, the HoeAc_2_Fl, a synthetic dye consisting of Hoechst 33342 and fluorescein diacetate moieties, was found to label nuclei of Arabidopsis guard cells. In contrast to the classical DAPI and Hoechst dyes, which are excited with the ultraviolet-range wavelength laser, HoeAc_2_Fl allowed excitation at a longer wavelength (Ex/Em, 488/520 nm), which is less phototoxic to cells. However, unfortunately, the staining of nuclei was only observed in closed stomata and not in opened stomata (Takaoka et al. 2020), and no information was provided for other types of tissue. SYTO12, one of the commercially available SYTO^®^ dye series, has also successfully labeled mitotic chromosomes in live male meiotic cells in maize (Yu et al. 1997, Nannas et al. 2016). Still, further investigations are required to see if this dye and HoeAc_2_Fl are also applicable to other cells and tissues. More recently, *N*-aryl pyrido cyanine (PC) derivatives have been developed, and the nuclei of Arabidopsis root and leaf tissue, including epidermal and mesophyll cells, were well stained by PC1 (Ex/Em, 532/546 nm) and PC3 (552/600 nm) (Uno et al. 2021). So far, PC dyes may be the best solution for in vivo imaging of plant nuclei.

### Cell walls

Plant cell walls are mainly composed of a diverse set of polysaccharides (Höfte and Voxeur 2017). Propidium iodide has been implicated in binding to the negatively charged portion of homogalacturonans in plant cell walls and is the most popular counterstaining dye in live-cell imaging for morphological studies (Rounds et al. 2011). Cellulose microfibrils are one of the key components of plant cell walls and are synthesized by cellulose synthase complexes bound to PM, which determines the direction of cell growth. Anderson et al. (2010) found that Pontamine Fast Scarlet 4B (alternatively known as Direct Red 23) effectively labeled cellulose microfibrils in Arabidopsis to structurally characterize the cell walls. Callose (β-1,3-glucan) is deposited in the cell wall near the neck zone of the plasmodesmata—microscopic pores that connect plant cells. Aniline blue, which stains callose, has been used to mark plasmodesmata in Arabidopsis and tobacco leaves (Kraner et al. 2016). The covalent cross-linking of rhamnogalacturonan II (RG-II), a pectic polysaccharide, which is mediated by boric acid, is essential for cell wall integrity. Dumont et al. (2016) successfully labeled RG-II using 3-deoxy-d-manno-oct-2-ulosonic acid (Kdo). Pulse labeling experiments of Kdo analog, Kdo-N_3_ (8-azido 8-deoxy Kdo), have revealed that RG-II synthesis was reduced in Arabidopsis root differentiation zone. A drawback of Kdo labeling is that it labeled only the cells closest to the surface of the tissue, possibly due to the limited penetrance of the fluorescent dye into deeper regions of the tissue. The secondary cell walls are often visualized noninvasively through lignin autofluorescence with standard green FP filter sets (Naseer et al. 2012). Since many autofluorescent compounds, which can emit fluorescence in the absence of external fluorescent markers, are found in plants and lignin fluorescence is sensitive to the molecular environment (Donaldson 2020), it may be useful to use a complementary method to visualize the secondary cell walls by incorporating fluorescently labeled monolignols into the lignin (Tobimatsu et al. 2013).

### Cell viability

In many assays, it is critical to test for cell viability, ideally with a simple staining method. Similarly, the analysis of cell-death-related processes requires dyes that can identify cells undergoing apoptosis (Reape and McCabe 2008). Trypan blue (excited at 620 nm and detected at 627–720 nm) and propidium iodide (excited at 535 nm and detected at 590–660 nm), which are commonly used to stain cell walls (Mou et al. 2000, Rounds et al. 2011), cannot permeate the PM of intact cells and are thus used to identify viable and nonviable cells. The fluorescent SYTOX dyes, which bind to DNA, are also used to identify nonviable cells within living plant tissues (Truernit and Haseloff 2008). SYTOX green (Ex/Em, 504/523 nm), orange (547/570 nm) and blue (444/480 nm) are commonly used in Arabidopsis.

## Dyes for Monitoring Biochemical and Biophysical Processes and Signaling

Fluorescent probes are widely used for monitoring cellular processes such as signaling molecules and changes in pH, membrane potential or microviscosity (**Table 1**).

Reactive oxygen species (ROS) accumulate under stress conditions and function as stress responses in development and in environmental responses. ROS can be produced by enzymatic activities of several peroxidases (Suzuki et al. 2011). In vivo imaging of ROS has been achieved by ROS-responsive chemical probes, based on fluorescein derivatives (detected with Ex/Em at 485/535 nm). H_2_DCFDA, a non-fluorescent AM derivative of 2ʹ,7ʹ-dichlorodihydrofluorescein (H_2_DCF) injected into pathogen-infected tobacco leaves, was oxidized by ROS to produce fluorescent 2ʹ,7ʹ-dichlorofluorescein (DCF) (Govrin and Levine 2000). In addition to H_2_DCFDA, a chloromethyl derivative, CM-H_2_DCFDA, has also been employed for the visualization of intracellular ROS in plant cells (Monshausen et al. 2007, Fichman et al. 2019). Another commercially available ROS-responsive fluorescein derivative OxyBURST Green dihydro-2ʹ,4,5,6,7,7ʹ-hexafluorofluorescein (H_2_HFF) bovine serum albumin (BSA) has been used for extracellular ROS imaging, successfully monitoring extracellular ROS production during root hair apical growth and ROS accumulation in leaves under high light (Monshausen et al. 2007, Fichman et al. 2019). Comparison of H_2_DCFDA and OxyBURST Green H_2_HFF used for Arabidopsis whole-plant imaging showed that H_2_DCFDA had higher signal-to-noise ratio and cell permeability. Unlike the fluorescein-derivative ROS-sensitive dyes, Peroxy Orange 1 (PO1) (Dickinson et al. 2010) has photochemical properties similar to those of orange FPs (detected with Ex/Em, at 543/545–750 nm) and is thus compatible with green/yellow FPs. Although less responsive than PO1, other ROS-responsible dyes, such as Amplex Red (*N*-acetyl-3,7-dihydroxyphenoxazine) and dihydroethidium, have been used to observe ROS accumulation in leaves (Fichman et al. 2019).

BCECF (pKa 7.0), the most popular pH indicator derived from fluorescein, is suitable for near-neutral pH monitoring. For the use as a cytosolic pH indicator in plant cells, BCECF was fused with dextran and microinjected into cells. The dye was used in a ratiometric assay by exciting at two wavelengths, 450 and 490 nm, and collecting emission at 520 nm, and pH changes in the cytoplasm of lily pollen tube have been quantitatively analyzed (Feijo et al. 1999). 8-Hydroxypyrene-1,3,6-trisulfonic acid trisodium salt (HPTS; pKa 6.4) has also been used in ratiometric assays by excitation at two wavelengths, 405 nm and 458 nm, and detection of emission at 514 nm. Using HTPS, which is membrane impermeable, apoplasmic pH changes from pH 4.6 to pH 6.4 in Arabidopsis roots, hypocotyls and cotyledons were quantitatively analyzed (Barbez et al. 2017, Dünser et al. 2019). Seminaphthorhodaflours-1 (SNARF-1) shows varying emission intensities at wavelengths of around 580 nm and 640 nm, depending on the pH when excited by a 488-nm laser (Whitaker et al. 1991). The SNARF-1 can pass through the PM of tobacco BY-2 protoplast and cell wall and the PM of Arabidopsis suspension cells and monitor cytosolic pH changes (Clarke et al. 2005, Sano et al. 2010). In other work, Oregon Green (OG) (pKa 4.7; Ex/Em, 488/514 nm) covalently attached to the cellulose-binding domain (CBD) has been used as an apoplasmic pH indicator because of its pH-dependent change in fluorescence intensity in the acidic pH range. CBD-OG showed the pH change in the apoplasm of Arabidopsis root cells upon gravitropic stimuli (Fasano et al. 2001).

Membrane potentials can be monitored by microelectrodes, but voltage-sensitive dyes provide spatial information in intact organs and other molecules such as calcium ion can be measured simultaneously (Konrad and Hedrich 2008, Lu et al. 2016). The voltage-sensitive dye *bis*-(1,3-dibutylbarbituric acid)-trimethine oxonol [DiBAC_4_(3)] is transported to the cytoplasm in response to depolarization. DiBAC_4_(3) is utilized to monitor the membrane potential of single cells such as protoplast and pollen tube and mature plant tissue such as root epidermal cells (Konrad and Hedrich 2008, Dejonghe et al. 2016, Hoffmann et al. 2020). Recently, Hoffmann and co-workers used a voltage-sensitive dye ANNINE-6plus, which shows strong membrane binding and increased fluorescence due to membrane hyperpolarization, to show that decrease of PM H^+^-ATPases resulted in less hyperpolarization of the pollen tube (Hoffmann et al. 2020). ANNINE-6plus can be also combined with two-photon microscopies to observe the membrane potential in deeper tissues (Roome and Kuhn 2020). Proton gradients in mitochondria are monitored by MitoTracker™ dyes. Dejonghe et al. (2016) demonstrated that Endosidin9 and the endocytic inhibitor Tyrophostin A23 behave as mitochondrial uncouplers through monitoring the mitochondrial membrane potential using MitoTracker™ Red CM-H_2_XRos.

Spatial variations in microviscosity induced throughout the cells provide insight into local mechanobiological processes. It is thus crucial to elucidate intracellular microviscosity patterns to understand plant mechanobiology. Most recently, BODIPY-based molecular rotors (BDPs) have been developed, which enable the measurement of microviscosity when combined with the fluorescent lifetime imaging microscopy. This technique provided PM and cell wall microviscosity maps in epidermal cells of Arabidopsis root and leaf (Michels et al. 2020).

## Covalent Self-labeling Technologies in Plant Cells

Chemical dyes localize to compartments and molecules based on their chemical properties, but do not bind to specific proteins by themselves as the localization is merely a passive process. On the other hand, genetically encoded probes that make use of FPs can be targeted specifically to target domains of specific proteins. Covalent labeling technology provides a way to combine the advantages of synthetic fluorescent probes with genetical tagging. It complements and combines the benefits of existing labeling methods with synthetic dyes and FPs and offers advantages over conventional FPs, including access to a wide variety of synthesized and designed organic dyes and smaller size relative to standard FPs (27 kDa).

**Fig. 1:**
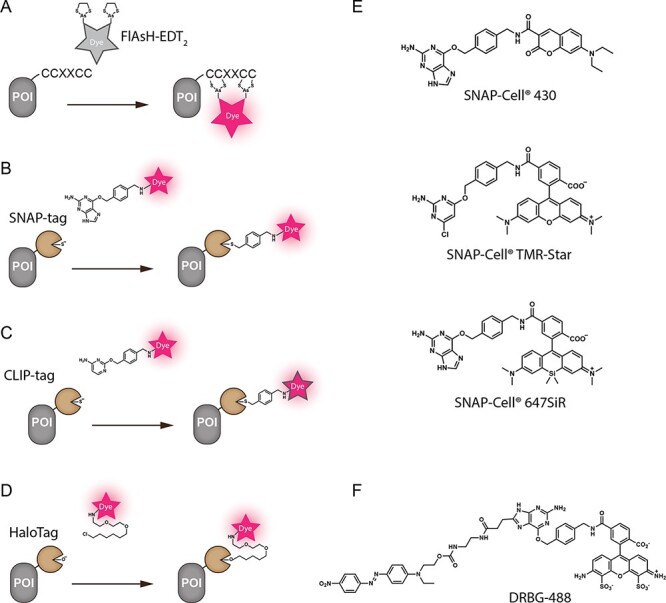
Schematic illustration of covalent protein labeling technologies and SNAP-tagging dyes. POIs are tagged with genetically encoded markers. (A) Non-fluorescent FlAsH-EDT_2_ covalently binds to TC sequence to form a fluorescent FlAsH-TC complexes. (B) SNAP-tag reacts with benzylguanine to form a covalent bond. (C) CLIP-tag reacts with benzylcytosine. (D) HaloTag reacts with chloroalkane ligand. (E) Cell-permeable SNAP-tagging probes used to label α tubulin (TUA5) proteins in plant cells (Iwatate et al. 2020). (F) Cell-impermeable SNAP-tagging dye used to label PIN2 proteins in Arabidopsis plants (Iwatate et al. 2020).

The ideal tag for covalent labeling should be as small as possible to reduce perturbation. Tetracysteine (TC) tag labeling has been developed based on the interaction between fluorescein arsenical helix binder-ethanedithiol (FlAsH-EDT_2_) and a 15-amino-acid small peptide motif containing the sequence CCXXCC (Griffin et al. 1998) (**Fig. 1A**). A non-fluorescent FlAsH-EDT_2_ is covalently linked to the TC motif and forms a strongly fluorescent complex. In Estévez and Somerville (2006), a synthetic glycopeptide (SynGMs) containing the arabinogalactan protein motif was fused with a TC tag and expressed in Arabidopsis plants. FlAsH-TC labeling allowed for tracking of the expression and localization of SynGMs in living cells; however, a weak nonspecific reaction was detected in the nuclei of Arabidopsis. To our knowledge, no other reports of FlAsH-TC being used in plant cells are available, so there is still a lack of understanding of their usability.

Kai Johnsson’s group has pioneered the development of a covalent self-labeling technique that efficiently binds small-molecule-based fluorescent probes to POIs in vivo (Keppler et al. 2003). SNAP-tag, in which the target protein is fused to the human DNA repair enzyme *O*^6^-alkylguanine transferase with a size of 20 kDa, labels proteins specifically by reacting with benzylguanine-conjugated dyes to form covalent bonds (**Fig. 1B**). The covalent labeling of SNAP-tag with *O*^6^-benzylguanine occurs through a well-defined reaction, achieved within 5–10 min in vitro and within 60 min in *Escherichia coli* (Keppler et al. 2003). Recently, we demonstrated that the covalent labeling of SNAP-tag with synthetic probes can also occur in live plant cells (5–60 min depending on the probes) and SNAP-tag technology is applicable to plant researches (Iwatate et al. 2020). Benzylguanine moiety has limited contribution to the hydrophilicity of the probe; thus, the selection of the probe for SNAP-tagging depends predominantly on the membrane permeability of the dyes. Permeable dyes are suitable for SNAP-tagging of cytoplasmic processes, while cell-impermeable dyes are suitable for tagging membrane proteins at the cell surface. SNAP-tagging dyes (SNAP-Cell^®^ 430, SNAP-Cell^®^ TMR-Star and SNAP-Cell^®^ 647-SiR), which showed plant cell membrane permeability using tobacco BY-2 cells (**Fig. 1E**), were used to label SNAP-tag tubulin in Arabidopsis. The time-lapse imaging of Arabidopsis root cell division revealed that SNAP-tagging did not exhibit significant cytotoxicity compared to FPs and demonstrated that it is feasible to use SNAP-tagging in combination with FPs for three-color live-cell imaging (Iwatate et al. 2020). One of the major advantages of the SNAP-tag system is the availability of a wide range of dyes. DRBG-488, a non-fluorescent cell-impermeable dye with an intramolecular quencher, only becomes fluorescent when covalently bound to the SNAP-tag (**Fig. 1F**) (Komatsu et al. 2011). This allows the fluorescence-activation-coupled protein labeling, resulting in a high signal-to-noise ratio. DRBG-488 attached to PIN2 at the membrane surface enabled the monitoring of endocytosis and intracellular trafficking of membrane proteins (Iwatate et al. 2020).

Other covalent tags such as CLIP-tag and HaloTag have also been developed to enable the simultaneous multicolor labeling of multiple proteins (Gautier et al. 2008, Los et al. 2008), which is worth exploring in plant systems. CLIP-tag (20 kDa) self-labels by reacting with *O*^2^-benzylcytosine (**Fig. 1C**), and HaloTag (33 kDa) utilizes a mutant bacterial dehalogenase that forms stable bonds with chloroalkane ligands (Los et al. 2008) (**Fig. 1D**). With further effort, such as the implementation of super-resolution imaging and the site-specific use of dyes to monitor biochemical and biophysical processes and signaling, this covalent self-labeling technology will prove to be useful for addressing exciting unexplored biological questions.

## Perspectives

Live-cell imaging is an important technology for plant cell biology, and the scope and values of the technology are closely tied to the availability of suitable fluorescent probes. A wide range of dyes available for plant cell biology research are shown in **Table 1**; however, there is still room for further development such as dyes used at near-infrared (NIR) wavelengths (650–900 nm). PREX710 (Ex/Em, 712/740 nm), the longest-wavelength dyes among those tested for membrane permeability using BY-2 cells, was not taken up to the cell (Iwatate et al. 2020). Therefore, PREX710 may be suitable for extracellular staining such as cell walls. The modification of PREX710 with a benzylguanine moiety would enable the visualization of SNAP-tagged membrane proteins in NIR. Increasing the membrane permeability may make the dye useful for labeling cytoplasmic processes. A frequently used method to increase the cell membrane permeability of dyes is to modify AM ester groups, which imparts hydrophobicity in chemical compounds (Tsien 1981). In plant cells, ester loading has achieved only limited success (Swanson et al. 2011). In recent decades, the application of cell-penetrating peptide (CPP) in chemical dyes has attracted much attention as a way to improve the delivery efficiency of molecules into plant cells (Numata et al. 2018). CPP technology has the potential to expand the range of applications, such as allowing membrane-impermeable synthetic dye to be used inside cells.

Self-labeling with chemical dyes via SNAP-tag can be effectively used in plant cells. In mammalian cells, a BODIPY-based calcium indicator has been successfully localized to specific compartments using SNAP-tagging to measure local calcium concentrations (Kamiya and Johnsson 2010). Covalent self-labeling is a promising technology that may open up new avenues for live-cell imaging in plant cells, including site-specific observation of biochemical and biophysical processes and single-molecule imaging. Recently, chemical probes that label themselves in a covalent manner according to enzymatic reaction have also been developed (Kwan et al. 2011, Doura et al. 2016). As these probes do not require gene expression, they could provide further advantages over current labeling systems.

## Data Availability

No new datasets were generated or analyzed in this study.
